# Variation in Phenolic Compounds and Antioxidant Activity of Various Organs of African Cabbage (*Cleome gynandra* L.) Accessions at Different Growth Stages

**DOI:** 10.3390/antiox10121952

**Published:** 2021-12-06

**Authors:** Sylvia Maina, Da Hye Ryu, Gaymary Bakari, Gerald Misinzo, Chu Won Nho, Ho-Youn Kim

**Affiliations:** 1 Convergence Research Center for Smart Farm Solution, Korea Institute of Science and Technology (KIST), Gangneung 25451, Korea; wairimusylvia@kist.re.kr (S.M.); dahye0507@kist.re.kr (D.H.R.); cwnho@kist.re.kr (C.W.N.); 2 SACIDS Foundation for One Health, Sokoine University of Agriculture, Morogoro 67125, Tanzania; gaymary.bakari@sua.ac.tz (G.B.); gerald.misinzo@sacids.org (G.M.); 3 Division of Bio-Medical Science and Technology, KIST School, University of Science and Technology (UST), Daejeon 34113, Korea

**Keywords:** orphan leafy vegetable, bioactive compounds, rutin, African cabbage, radical scavenging

## Abstract

The presence of nutritional and health-benefiting compounds has increased awareness of orphan leafy vegetables such as *Cleome gynandra* (CG), whose phytochemicals vary among accessions and organs during growth. This study investigated the polyphenol accumulation and antioxidant activities (AOA) of eight CG accessions from the vegetative stage to the seed set stage. Plants were separated into leaves and stem (LS), flowers, and silique organs, and extracts were analyzed for total phenolic content (TPC), total flavonoid content (TFC), rutin and astragalin content, and AOA using 2,2-diphenyl-1-picrylhydrazyl-hydrate (DPPH) and 2,2′-azino-bis(3-ethylbenzothiazoline-6-sulphonic acid) (ABTS). There were significant interaction effects of growth stages and accessions that contributed to changes in compounds content and AOA. TPC accumulated in plant generative parts, whereas flavonoids accumulated in young plant organs. HPLC profiling revealed that rutin was the most abundant compound in all organs, with flowers having the highest levels, while astragalin was only found in flowers. Silique extracts, particularly accession KF-14, recorded the highest TPC, which corresponded to the strongest radical scavenging activity in ABTS and DPPH assays and a strong linear correlation. The germplasm contained accessions with significantly different and varying levels of bioactive compounds and AOA. These findings potentiate the exploitation of CG organs such as siliques for AOA, flowers for rutin and astragalin, and young shoots for flavonoids. Moreover, the significant accumulation of the compounds in particular accessions of the germplasms suggest that such superior accessions may be useful candidates in genetic breeding programs to improve CG vegetable.

## 1. Introduction

*Cleome gynandra* L. (CG), a member of the *Cleomaceae* family of vegetable crops, is native to the tropical and subtropical regions of Africa and Asia, where it grows in spontaneous flora or in volunteer crop and is also semi-cultivated as a vegetable [1,2,3,4]. The tender leaves, young shoots, and flowers of this “super vegetable” are used as potherb, salads, stews, and flavors [2,5]. CG extracts and preparations have long been used in traditional medicine to treat wounds, fevers, headaches, earaches, eyes, marasmus, scurvy, gastrointestinal disorders, diabetes, and childbirth induction [5,6].

Due to their abundant secondary metabolites with therapeutic properties, plants have emerged as a focal point in the search for safe natural and alternative compounds to prevent emerging and reemerging diseases and health conditions [7,8]. These metabolites include glucosinolates, phenolic, and flavonoid compounds, which confer other traits to the plant such as defense mechanisms, pollinator attractants, and accumulation in response to stress [9,10]. Leafy vegetables, which account for a large portion of the global human diet, have been identified as a particularly rich and essential source of such metabolites [11]. Research shows that a high vegetable diet is associated with an improved total antioxidant status of blood and cellular antioxidant activities, as well as lower risks of associated diseases such as cardiovascular disease, whereas a low vegetable intake is associated with risks and predisposition to heart diseases [11,12,13].

The nutritional and non-nutritional benefits of vegetables, including African indigenous vegetables (most of which are still largely unrecognized), are increasing their current demand, resulting in increased interest from consumers, researchers, and dieticians [2,4]. Phenol compounds are among the most abundant and well-studied secondary metabolites in leafy vegetables, being associated with numerous health benefits [14], and CG polyphenol levels are reported to be comparable to other African vegetables such as African nightshade, tree spinach, and burning nettle, most of which are associated with a variety of health benefits [15].

In previous studies on the phytochemicals and food quality of CG, some beneficial compounds that were identified include phenolic compounds (caffeic, coumaric, sinapic, hydroxycinnamic acids; ferulic acid; quercetin; and kaempferol derivatives) [4,16,17,18], glucosinolates, steroids, tannins, and carotenoids [17,19,20]. Scientific investigations have also reported the antimicrobial, anti-inflammatory, anticancer, analgesic, antioxidant, and antidiabetic activities of CG [1,21,22,23,24,25,26]. Compounds such as quercetin, catechins, dihydroquercetin, and rutin are reported to be excellent sources of natural antioxidants in most green leafy vegetables, including spinach, kale, collard greens, lettuce, purslane, amaranthus, and sweet potato greens [27,28].

The polyphenols composition in plant organs varies quantitatively and qualitatively, with some such as catechins and quercetins being ubiquitous in vegetables and some others being restricted to specific species [29]. Furthermore, factors such as plant phenology and changes in environmental conditions, which influence the compounds’ biosynthetic pathways, influence the content, composition, and biological activities associated with polyphenols [30,31]. Plants such as purslane [32], amaranth [33], and sage [34] have shown such changes in phenolic profiles and their associated antioxidant activity at various phenological stages.

With the shift in food consumption over the decades, there is a demand for foods with more functionality [35]; thus, studies are being conducted to identify superior cultivars/germplasms, as well as to establish optimal cultivation conditions and proper harvest timing. However, information on proper harvesting time, superior cultivars, and how compounds vary in plant organs and accessions at different growth stages with effects on the antioxidant capacity of plant extracts is lacking in CG. This study, therefore, aimed at profiling the phenolic compounds and evaluating the antioxidant potential of extracts from organs of eight accessions of CG collected at vegetative, flowering, and seed set stages of the plants. The results from the current study will help improve our understanding of the variability and accumulation of polyphenols and the antioxidant potential of the vegetable to enable the choice of favorable organ and stage to be applied in the food and pharmaceutical industries. Similarly, the evaluation of these compounds among several accessions of a plant is essential to help breeders in selecting the appropriate accession for use in improving the leafy vegetable.

## 2. Materials and Methods

### 2.1. Materials

For the assay and analysis, anhydrous sodium carbonate, sodium nitrite, sodium hydroxide, and aluminum chloride hexahydrate were obtained from Duksan Pure Chemical Co. (Ansan, Korea), and 2,2 diphenyl-1-picrylhydrazyl ethanol (DPPH) and 2,2′-azino-bis(3-ethylbenzothiazoline-6-sulphonic acid) (ABTS), Folin–Ciocalteu phenol reagent, gallic acid (CAS No. 149-91-7), rutin (CAS No. 205-814-1), and kaempferol-3-glucoside (CAS. No. 480-10-4) were obtained from Sigma-Aldrich (St. Louis, MO, USA). All solvents used for HPLC analysis were HPLC-grade.

### 2.2. Plant Material and Cultivation Conditions

Seeds of eight different accessions (TT-00, UAG/1907C, ELG/1907C, ELG/1907B, WPK/2007, KF-14, KF-05A, KF-03) of CG were obtained from the Centre for Biodiversity Kenya Resources Centre for Indigenous Knowledge, National Museums of Kenya, and germinated in a growth chamber at the SMART FARM in KIST (Gangneung, Korea). The seeds were sown in 200 holed trays with soil at a pH of 5–7, volume density = 0.3, and E.C ≤ 1.0 ds/m at a temperature ranging between 25 and 30 °C, humidity 60–80%, and 16/8 h day/night condition. After 1 week, the germinated plants were transplanted to pots and transferred to the greenhouse, whose temperature conditions were maintained at 20–25 °C. Sampling was done at vegetative, flowering, and seed set stages of the plant, and the various organs of the sampled materials were separated into roots, flowers siliques, and a combination of leaves and stem (LS). Samples were immediately flash-frozen in liquid nitrogen and stored at −80 °C until lyophilization in the freeze-drier for 4 days. The experiment was executed in a completely randomized blocked design.

### 2.3. Preparation of Ethanol Extracts

A total of 200 mg of finely ground powder of the various collected organs were prepared in triplicate, dissolved in 4 mL ethanol (70%), and extracted twice in a sonicator (Bandelin Sonorex, Berlin, Germany) set at 40 °C for 15 min each [13]. The extracts were centrifuged at 3000 RPM for 15 min at 4 °C and at a rotation radius of 325 mm. The supernatants were filtered through a 0.45 μm pore size filter membrane then concentrated under a stream of nitrogen gas to dryness. Concentrated dry samples were dissolved in dimethyl sulfoxide (DMSO) to 20 mg/mL and filtered through a filter membrane of 0.22 μm pore size, then stored at −80 °C until further analysis.

#### 2.3.1. Determination of Total Phenolic Compounds Contents (TPC)

TPC was determined using the Folin–Ciocalteu method with some modifications [36]. A total of 10 μL of plant extract (4 mg/mL) or gallic acid standard in triplicate was mixed with 100 μL of sodium carbonate (Na_2_CO_3_, 2%) solvent in a 96-well microplate and agitated for 3 min; then, 10 μL of 1 N Folin reagent was added to the mixture, and the plates were incubated at room temperature in dark for 30 min. The TPC was determined by measuring the optical density of the plates with a multi detection microplate reader (Synergy HT; BioTek Instruments, Winooski, VT, USA) set at a wavelength of 750 nm. TPC was calculated on the basis of a calibration curve determined using gallic acid (0.010–0.625 mg/mL) and was expressed in milligram gallic acid equivalents (GAE) per gram of the extract dry weight (DW) with the calibration curve y = 0.9084x + 0.0101 (R^2^ = 0.9997), where y represents the absorbance detected at 750 nm, and x represents the content in gallic acid equivalents (mgGAE/mL). The calculated content was converted to mgGAE/g by considering the dilution factor.

#### 2.3.2. Determination of Total Flavonoid Content (TFC)

The TFC was determined using the colorimetric aluminum chloride (AlCl_3_) assay method previously described [37], with minor modifications. Briefly, 10 μL of sodium nitrite (NaNO_2_, 5%) was added to a 10 μL sample extract (4 mg/mL) in a 96-well microplate and allowed to react for 6 min at room temperature away from light. The mixture was then reacted with 20 μL of aluminum chloride (AlCl_3_.6H_2_O, 10%) for 5 min before we added 50 μL of sodium hydroxide (NaOH, 1M) and 110 μL of distilled water. After 10 min incubation, TFC was determined by measuring absorbance at 510 nm with a multi detection microplate reader (Synergy HT; BioTek Instruments, Winooski, VT, USA). In this experiment, rutin (0.16–5.0 mg/mL) was used as the standard for the development of the calibration curve as follows: y = 0.0291x + 0.0034 (R^2^ = 0.9995), where y represents the absorbance detected at 510 nm, and x represents the flavonoid content (mgRE/mL). The total flavonoids were expressed in milligram rutin equivalent (RE) per gram of extract dry weight (DW) using the dilution factor.

#### 2.3.3. Determination of DPPH Antioxidant Activity

The capacity to scavenge free radicals was measured colorimetrically using the previously described DPPH de-colorization method with minor modifications [38]. In a 96-microwell plate, 190 μL of freshly prepared ethanolic solution of DPPH (0.2 mM) was mixed with 10 μL of extracted samples prepared in graded concentrations ranging from 1 mg/mL to 20 mg/mL. After 30 min of incubation at room temperature in the dark, the absorbance of the reagent solution was measured with a multi-detection microplate reader (Synergy HT; BioTek Instruments, Winooski, VT, USA) set to 517 nm. The following equation was used to calculate the percent (%) inhibition of the DPPH radical [37].

% inhibition = (1 − (AS/AC)) * 100 (AC is the absorbance of the control (ethanol and DPPH reagent) and AS is the absorbance of the tested extract sample).

#### 2.3.4. Determination of ABTS Antioxidant Activity

To make the antioxidant reagent, we reacted a 2,2-azino-bis(3-ethylbenzothiazoline-6-sulfonate) (ABTS) tablet dissolved in water with 2.45 mM potassium persulfate solution to achieve a concentration of 7 mM, and then incubated the mixture overnight at 4 °C in the dark to generate free radicals. The antioxidant assay was performed as described in a previous protocol with minor modifications [39]. Briefly, 10 μL of extracted samples prepared in concentrations ranging from 0.1 to 20 mg/mL in a 96-microwell plate were reacted with 190 μL of the ABTS^+^ reagent. The plates were incubated in the dark for 5 min before the absorbance was measured at 734 nm using a multi detection microplate reader (Synergy HT; BioTek Instruments, Winooski, VT, USA). The % radical scavenging activity of the extracts DW was calculated as described above [37].

% inhibition = (1 − (AS/AC)) * 100 (AC is the absorbance of the control, a mixture of ethanol and ABTS reagent and AS is the absorbance of the tested extract sample).

#### 2.3.5. Quantification of Bioactive Compounds

Individual phenolic compounds were analyzed using an Agilent HPLC system (Agilent 1260 series, Santa Clara, CA, USA) that consisted of a degasser, quaternary pump, an autosampler, and a diode array detector (DAD), as well as ChemStation software. For analysis, dry extracts samples were dissolved in DMSO to 10 mg/mL. A total of 10 μL was injected at a flow rate of 0.8 mL/min into a YMC-Triart C18 column (250 × 4.6 mm, 5 µm) maintained at 35 °C. The binary mobile phase consisted of 0.2% formic acid (FA) in water (*v/v*) (solvent A) and 0.2% FA in acetonitrile (*v/v*) (solvent B). The solvents were maintained in the following separation: 0–1 min (0.0–11.0% B), 1–18 min (11.0–11.1% B), 18–19 min (11.1–37.8% B), 19–32 min (37.8% B), 32–33 min (37.8–82.0% B), 33–40 min (82.0–100.0% B), 40–43 min (100.0–50.0% B), and 43–46 min (50.0–0.0% B). A post-run option in the HPLC machine was employed at the end of each sample run to recondition the column to stable conditions. The DAD data were acquired at 280 nm for the different phenolic compounds. Quantification of known identified compounds was done using their external standards-specific calibration curves.

For the quantification, peak areas from the extract samples were analyzed using three replications and various concentrations of the external standards: quercetin-3-*O*-rutinoside (100–1000 µg/mL) and kaempferol-3-*O*-glucoside (0.5–20 µg/mL), which were analyzed in considering the minimum and maximum peak areas from samples [34]. The quercetin-3-*O*-rutinoside (rutin) quantification was conducted using the formula y = 8.1476x − 52.4480, R^2^ = 0.9998, while for kaempferol-3-*O*-glucoside, quantification was conducted using the formula y =14.784x + 8.6949, R^2^ = 0.9990, where y represents the peak area (mAU), and x represents the content (µg/mL). Then, the calculated compound content was converted into the mg/100 g by multiple dilution rate. Quantified compounds were expressed in milligram per gram of the extract’s dry weight.

### 2.4. Statistical Analysis and Correlation Analysis

All data are expressed as the mean ± standard deviation for three independent replicates used in the experiments. A one-way analysis of variance (ANOVA) was used to determine significant differences between the means of the experimental groups of the accessions, and a two-way ANOVA was used to analyze for significant differences and determine interaction effects of the growth stage and accessions factors in the agricolae package in R [40]. Post hoc analysis with Tukey’s multiple comparison test was performed to determine the specific groups responsible for the significant differences. Before ANOVA, the normality of residuals and equality of variances were checked by plotting models using plot type 2 and tested with a Shapiro test. To evaluate the relationship between the antioxidant activities and the different compounds, we performed Pearson correlation coefficient analysis and principal component analysis (PCA) in R. In the analysis, 95% confidence level or a probability less than *p* < 0.05 was considered as statistically significant.

## 3. Results

### 3.1. Total Phenolic Contents and Total Flavonoid Contents

TPC and TFC levels in various organ extracts varied during the vegetative, flowering, and seed set stages, with TPC displaying a different pattern compared to TFC, as shown in the summary content of the accessions (Table 1). Generally, TPC accumulated in the plant’s generative organs (flowers and siliques), whereas TFC was observed to be highest in the LS organs of the young plants. TFC levels also increased in the generative organs during the growth process. The silique extracts had the highest TPC levels, followed by the flowers, while the LS organs generally had lower contents, with LS at the flowering stage having the lowest content at 26.23 ± 5.41 mg GAE/g DW. TFC levels were highest in the LS organs during the vegetative growth stage and lowest in the LS organs during the seed set stage. The highest TPC in silique was 2.30-fold that of the lowest in the LSF, while the highest TFC in LSV was 1.34-fold that in LSS organs.

**Figure 1:**
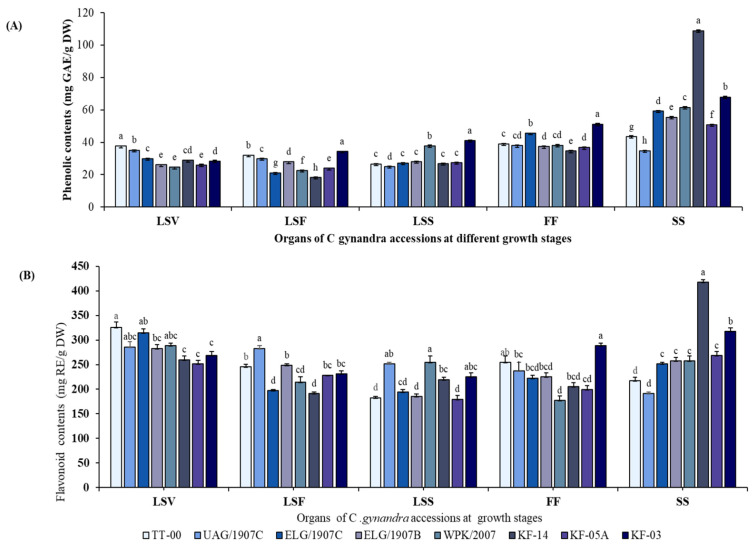
Changes in total phenolic content in mg GAE/g DW (**A**) and total flavonoid content mg RE/g DW (**B**) in the different organs of eight CG accessions during growth. Column and error bars represent the means and standard deviation (*n* = 3). Columns with different letters for each specific organ are significantly different (*p* < 0.05). mg GAE/g DW = milligram gallic acid equivalent per gram of dry weight; mg RE/g DW = milligram rutin equivalent per gram of dry weight; LSV = leaves and stem in the vegetative stage; LSF = leaves and stem in the flowering stage; LSS = leaves and stem in the seed set stage; FF = flower in the flowering stage; SS = silique in seed set stage.

Furthermore, the eight accessions studied had significantly different levels of phenolic and flavonoid in the various organs (Figure 1A,B). In addition, while the pattern of change for both TPC and TFC in the majority of the accessions organs during growth was remarkably similar, the siliques of accession KF-14 had a uniquely high and significantly different content of these compounds than the other accessions. In particular, accession KF-14 had the highest phenol content in silique extracts, which was 5.97-fold that of the flower extracts.

### 3.2. Changes in Organs AOA during Growth

**Figure 2:**
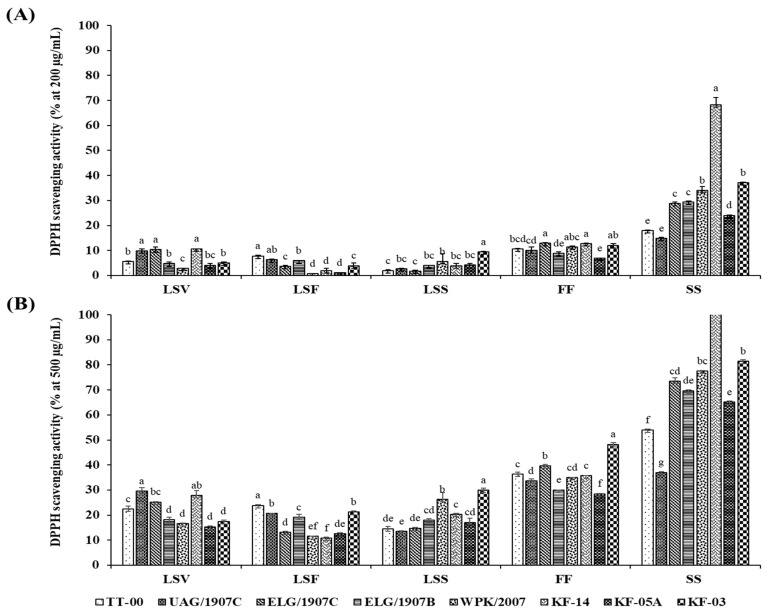
Dose-dependent (**A**,**B**) DPPH**^+^** radical scavenging activity of extracts from 8 accessions of CG organs collected at various stages. Columns with the different letters for each organ are significantly different (*p* < 0.05). LSV = leaves and stem in the vegetative stage; LSF = leaves and stem in the flowering stage; LSS = leaves and stem in the seed set stage; FF = flower part in the flowering stage; SS = silique part in the seed set stage.

**Figure 3:**
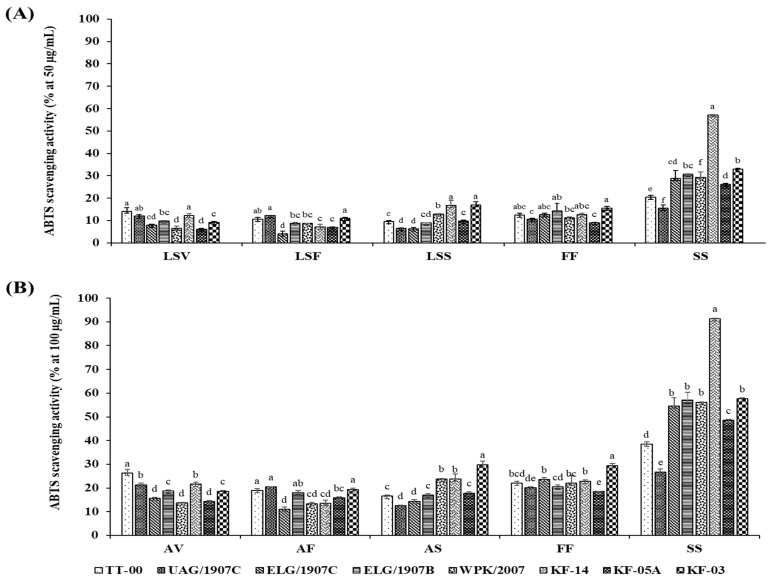
Dose-dependent (**A**,**B**) ABTS^•+^ radical scavenging activity of extracts from 8 accessions of CG organs collected at various stages. Columns with the different letters for each specific organ are significantly different (*p* < 0.05). LSV = leaves and stem in the vegetative stage; LSF = leaves and stem in the flowering stage; LSS = leaves and stem in the seed set stage; FF = flower part in the flowering stage; SS = silique part in the seed set stage Ability to scavenge free radicals expressed as RC_50_ values of CG accessions different organ extracts obtained at different growth stages.

The in vitro AOA was determined using the stable DPPH free radical scavenging activity and the radical cation ABTS scavenging capacity assays. Scavenging activities were ranked in the following descending order: SS > FF > LSS > LSV > LSF. Generally, the growth stage and organ type had a significant influence on the AOA, which also differed significantly among the accessions studied (Figure 2 and Figure 3). Moreover, although the pattern of change in AOA was consistent for both the DPPH and ABTS assays, the absolute values of the assays differed, with ABTS yielding higher scavenging activities for extracts at lower concentrations than DPPH assay. In particular, the highest and lowest percentage inhibitions exhibited by ABTS assay at extract concentration of 50 µg/mL were 57.22 ± 0.69% and 4.08 ± 1.09%, respectively, while DPPH exhibited the highest of 68.26 ± 2.96% and lowest of 0.71 ± 0.05% inhibition for sample concentration of 200 µg/mL.

Significant concentration-dependent inhibition of the DPPH and ABTS^+^ radicals was observed in all the tested extracts with a range between 0.74 ± 0.05% and 68.26 ± 2.96% at 200 µg/mL and between 10.85 ± 0.39% and 160.4 ± 6.51% at 500 µg/mL of extracted samples for DPPH activity, whereas in the case of the ABTS activity, the percentage inhibition ranged between 4.08 ± 1.09% and 57.22 ± 0.69% for 50 µg/mL, and 10.97 ± 1.49% and 91.35 ± 0.67% for 100 µg/mL of extracted samples. Notably, following the evaluation of AOA in the various accessions using both DPPH and ABTS assays, the silique extracts of accession KF-14, in particular, displayed a variation of approximately two- to fivefold higher inhibition compared to siliques of other accessions, indicating that the accumulation of the phenolic compounds that also influences the associated biological activity is genotype-dependent.

The estimation of the dose required scavenging fifty percent of radicals (RC_50_), and concentration was determined using results for graded series of sample concentrations; the results are summarized in Table 2. The lowest average RC_50_ value (396.71 ± 3.60 µg/mL) was recorded in the siliques, and the highest average value (1427.09 ± 13.17 µg/mL) was recorded in the LSF organs. The low RC_50_ in the siliques indicate that these extracts were more potent at low concentration compared to the LS extracts that required higher concentration to quench 50% of the radicals. Specifically, in the DPPH assay, the extracts efficacy and specific RC_50_ values for the accessions ranged between 137.27 and 721.45 µg/mL, 618.98 and 828.89 µg/mL, 855.62 and 1677.79 µg/mL, 1083.33 and 1877.51 µg/mL, and 925.95 and 1466.6 µg/mL for the extracts from siliques, flower, LSS, LSF, and LSV, respectively.

In the ABTS assay, the silique extract RC_50_ average of 110.34 ± 1.55 µg/mL had a threefold higher antioxidant activity compared to the lowest LSF extracts whose RC_50_ average was 352.12 ± 4.31 µg/mL. Specifically, the RC_50_ values for the different accessions ranged between 49.72 and 224.98 µg/mL, 179.03 and 295.26 µg/mL, 227.39 and 380.84 µg/mL, 271.68 and 472.95 µg/mL, and 218.94 and 399.47 µg/mL for the SS, FF, LSV, LSF, and LSS extracts, respectively.

### 3.3. Quantitative Analysis of Compounds from Extracts of Different Parts of CG

HPLC-DAD technique was applied to analyze the contents of quercetin-3-*O* rutinoside (rutin) and kaempferol-3-*O*-glucoside (astragalin) identified in the CG extracts using their specific standards calibration curves. The findings from this study are the first to detail how the leaves and stem, flowers, and siliques of CG differ quantitatively in the levels of rutin at vegetative, flowering, and seed set stages (Table 3), as well as how the variation occurs in various accessions of the plant (Figure S1). There was a significant interaction effect between the growth stages and the different accessions of CG that contributed to the variations in the content of rutin. Overall, the amount of rutin in the flowers for most of the accessions was considerably higher (ranging from 56.23 ± 0.63 to 92.00 ± 1.21 mg/g) and significantly different (*p* < 0.05) compared to other organs. The lowest amount of rutin was observed in the LSF organs with ranges from 25.81 ± 1.71 to 40.49 ± 0.80 mg/g in the various accessions. Moreover, although levels of rutin were generally low in the LS organ extracts, we observed higher levels at the mature stage compared to the vegetative and flowering stages. In addition, significant variations in the contents of rutin were noticed among the specific accessions and the pattern of change appeared different for each specific accession across the growth stages except for accession KF-14, which maintained significantly higher content throughout its growth.

Kaempferol 3-*O*-glucoside was only identified and quantified in the flower extracts, and its values ranged between 0.54 ± 0.27 and 2.02 ± 0.42 mg/g DW (Table 3). The ELG/1907B accession had significantly higher levels of astragalin than the other accessions, which did not show any significant differences in their content.

### 3.4. Correlation between Compounds and AOA

**Figure 4:**
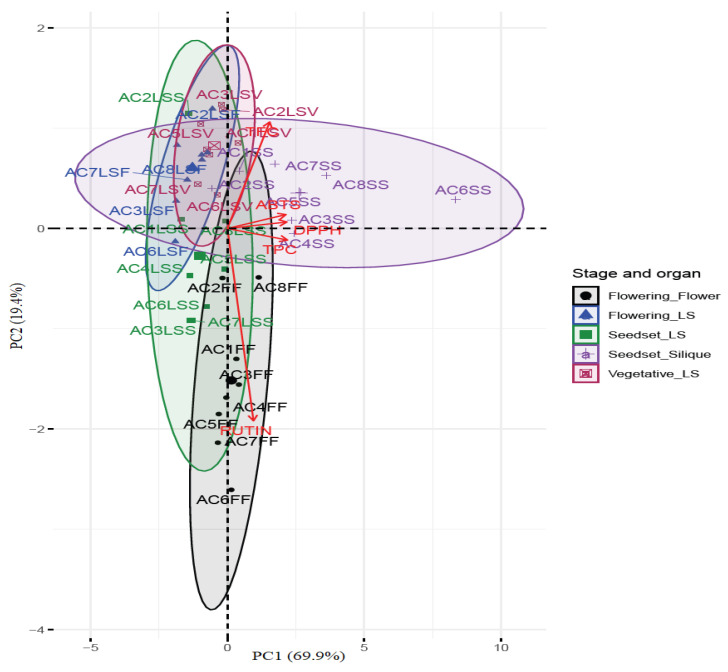
Principal component analysis (PCA) biplot of scores and loadings of the total phenolic, total flavonoid, and rutin content and antioxidant activities of extracts from organs at different growth stages of the eight accessions of CG. AC = accession, TFC = total flavonoid content, TPC = total phenolic content, ABTS = azinobis (3-ehtylbenzothiazoline-6-sulfonic acid), DPPH = 2,2 diphenyl-1-picrylhydrazyl, FF = flower, LSF = leaves and stem at flowering stage, LSS = leaves and stem at seed set stage, LSV = leaves and stem at vegetative stage, SS = siliques at seed set stage. AC1 = TT-00, AC2 = UAG/1907C, AC3 = ELG/1907C, AC4 = ELG/1907B, AC5 = WPK/2007, AC6 = KF-14, AC7 = KF-05A, AC8 = KF-03.

Principal component analysis (PCA) was performed to investigate the overall similarities and differences between rutin, TPC, and TFC compounds and their relationships to the antioxidant potential of the different organs of CG at the various stages of sampling. The variables on the PCA biplot shown in Figure 4 include the various phytochemicals and assays used in the study, which were mapped using loadings and scores in dimensional spaces determined by principal components. The principal component 1 (PC1) explained 69.9% of the variances, while (PC2) explained 19.4%, for a total of 89.3% of the variances, representing a linear combination of variables in most organs extracts. The silique extracts of most accessions were characterized by high TPC content and high AOA, while the FF extracts were characterized by high rutin content, and medium TPC and AOA.

Furthermore, the Pearson correlation analysis showed both significant positive and negative correlations between the compounds, AOA, and the RC_50_ values (Figure S4). Overall, we observed a strong positive correlation between TPC and DPPH, TPC and ABTS, and DPPH and ABTS variables at R^2^ = 0.96, 0.95, and 0.97, respectively, at *p ≤* 0.001, indicating that these variables have positive linear relationships. Moderate positive correlation was observed for the rutin content to TPC, TFC, DPPH, and ABTS at R^2^ = 0.44, 0.04, 0.34, and 0.29, respectively. Strong negative correlations were observed between the TPC and the RC_50_ values, indicating the contribution of TPC in enhancing extracts’ ability to scavenge free radicals at low concentrations.

## 4. Discussion

In this study, we report for the first time the variation of polyphenol compounds in various organs of CG accessions at different growth stages and how the changes affect overall antioxidant activities. Given that plants have been shown to have peak levels of secondary metabolites such as polyphenols [41,42], quantitative measurements and profiling of rutin and astragalin, as well as TPC and TFC estimation, were performed. TPC levels in this study ranged between 18.23 and 108.86 mg GAE/g DW, which was higher than previous reports from CG accessions, which ranged between 9.86 and 12.21 mg GAE/g DW [17]. Such variations are to be expected considering that the previously reported values were obtained from leaves and that several other factors such as the type of organ evaluated; biological and genetic diversity; and environmental, soil, seasonal, and year-to-year variations cause phytochemicals in vegetables to vary [43].

Previous research found that both developmental stages and plant genotypes affect phenolic levels in plant organs [44,45,46,47,48,49]. Such variations are consistent with our findings, in which we found significant TPC variations among CG accessions, with siliques having the highest levels, followed by flowers, and the LS organs having the lowest TPC during the flowering stage. We hypothesize that the low level of phenolic compounds in organs of younger plants is related to the low amount of lignin precursors in the cell wall, as a result of low tissue lignification in young plants [50]. TPC accumulated in plants’ generative organs (flowers and siliques), which is consistent with other studies that reported similar findings [42], and while the pattern of change for TPC was remarkably similar in the majority of the accession organs during growth, the siliques of accession KF-14 had a significantly higher content, indicating that genotype significantly influences the accumulation of the compounds as previously reported in broccoli [51].

Flavonoids have been shown to accumulate primarily in young plants and decrease during the flowering stage when the plant actively undergoes more differentiation other than metabolite synthesis [52]. This is consistent with our findings, which showed that LS organs in the vegetative stage had the highest TFC levels, which decreased during growth. The observed reduction of TFCs could be attributed to increased cell differentiation during the flowering stage, as well as other factors such as plants using flavonoids for defense, pollination, and reproduction [53,54,55]. Moreover, we noticed in this study, flavonoid biosynthesis and accumulation occurred independently in each plant organ [56], with variations in their contents depending on the growth stage of plant growth [57] and the plant genotype, as shown in broccoli accessions [51,58].

In the case of rutin (quercetin-3-rutinoside), previous reports show that it accumulates in the leaves of CG [17,18] with little information reporting the compound in the other plant organs or reporting the effect of growth stages on its levels. We found significant differences in rutin levels among plant organs at different growth stages, with the highest amounts in flowers and the lowest amounts in LS organs at the flowering stage, averaging 1.86- to 2.22-fold in the different accessions. There was also genotype variation among the CG accessions, with KF-14 maintaining generally high levels of rutin in its organs throughout the growth period, while the flowers of accession ELG/1907B had the highest astragalin levels. Similar findings for rutin variations were realized in buckwheat crops and caper bush, where the flowers displayed the highest contents and the young leaves displayed higher contents when compared to more developed ones [59,60]. The fluctuation and variation in rutin content in plant organs, cultivars, and stages can be attributed to differences in the expression of flavonoid biosynthesis genes with plant growth stages [60,61,62]. In buckwheat, for instance, the expression of the chalcone synthase gene was positively correlated with the highest rutin content in flowers [60], whereas the flavonol synthase-1 gene expression was responsible for rutin accumulation in its seeds [63].

African leafy vegetables high in phenolic compounds have been linked to high antioxidant activity and are thus of great interest for human consumption as well as traditional medicine [18]. The aromatic structures of phenolic compounds facilitate the structure–antioxidant activity relationship, and the presence of hydroxyl groups allows them to donate free hydrogen atoms to free radicals, effectively deactivating them [64]. As a result, the presence of these compounds in plants usually indicates their antioxidant potential [65], with high antioxidant activity being associated with increased hydrogen transfer to free radicals, which increases the inhibitory effects of extracts rich in phenolic compounds [66,67]. In our findings, the patterns of antioxidant activities evaluated by DPPH and ABTS are comparable to the content of phenolics, implying that these compounds were the main antioxidant in the two assays, and increasing their concentration in the extracts resulted in increased activity, as demonstrated by the dose-dependent antioxidant potential. The similarity in inhibition ranking order and scavenging activity pattern suggests that the antioxidant activity examined with both assays is mediated by similar mechanisms.

In the case of the LS extracts, the low inhibition percentage recorded could be attributed to the stem part, which was combined with the leaves to make the LS samples. Previous studies found that stem exudates contain relatively few phenolic compounds and that there is limited free flow of phenolic compounds in phloem tissue channels due to reactions that oxidize phenolic compounds, resulting in low phenolic contents [42]. Moreover, while we established that rutin was the most abundant compound in CG flowers, its content had only a moderate correlation with antioxidant activities. This could imply that phenolics among other plant metabolites [68], which have the highest antioxidant capacity, are responsible for the stronger AOA activity in CG.

When compared to the DPPH assay, the ABTS assay demonstrated better and higher radical scavenging activity at lower extract concentrations. A further determination of the RC_50_ values revealed that each accession’s organ extract at specific stages was approximately three times more effective with ABTS than with the DPPH assay. Assessing the antioxidant activity of sample extracts using various methods has revealed that different radicals react differently to different antioxidant compounds [69]. ABTS has been shown to better reflect the hydrophilic, lipophilic, and high pigmented antioxidants in various extracts than the DPPH assay [70]. The ABTS assay is also reported as a more sensitive method for determining antioxidant activity due to its faster reaction kinetics and high response to antioxidants [71]. As a result, this method could be used to evaluate and report the actual antioxidant potential of CG organ extracts. The antioxidant potential of the various CG accessions varied significantly, according to our findings. These variations could be attributed to changes in phytochemical accumulation in the growth stages, accessions, and organ extracts. Variations in antioxidant activity have been observed in Amur silver grass [49], spearmint, and horsemint [67]. Such a study demonstrating genotypic variation in phytochemicals provides useful information for breeding crops with desired traits.

TPC and TFC compounds have previously been found to have strong positive correlations with antioxidant activity in various studies [65,67]. The relationships derived in most of such findings largely relate a specific abundant compound to the biological potential and the contribution in the levels of metabolites. In this study, however, rutin only established a moderate correlation with the AOA and a weak correlation to the TFC in the extracts. These findings agree with a study in broccoli varieties that revealed that there is not always a clear relationship between the accumulation of a specific flavonoid compound and the total flavonoid content [58]. In the mature stages of the vegetable, we found a significant accumulation of polyphenols, whose levels were strongly correlated with antioxidant activity. These interesting findings show that although most edible leafy vegetables are harvested at early stages due to their appealing texture and good visual qualities, harvesting CG siliques could have alternative use as an antioxidant in the pharmaceutical industry.

## 5. Conclusions

In the present study, we evaluated the quantitative and qualitative variabilities in compounds composition, contents, and biological activity among various accessions and organs of CG vegetables at the different growth stages. Rutin was found to be the most abundant compound in all the organs, with the highest accumulation in the flowers, and its levels were found to be significantly and positively correlated to the TPC levels. The generative organs (siliques and flowers) of CG accessions accumulated more TPC, while the vegetative organs accumulated more TFC, and a linear relationship existed between the TPC, TFC, and the antioxidant activities evaluated through DPPH and ABTS assays. Most accessions of the plant exhibited variation in the levels of the phytochemicals and their related biological activities with accession KF-14 having distinctively high TPC, rutin, and AOA activity. The substantial variations in the phenolic compounds and overall AOA may provide valuable information on siliques as the choice of plant material to be used as a source of antioxidant compounds and flowers as a source of rutin and astragalin. Furthermore, on the basis of the different content of the compounds at different stages and accessions, plant breeding programs could exploit these findings to develop varieties with improved target compound contents as well as with increased biological activities. To further improve and increase the usefulness of CG, we recommend further studies to identify the genes responsible for controlling the beneficial phytochemicals and to determine how the variations occur in the various organs of the plant during the growth process.

## Figures and Tables

**Table 1:** Total phenolic and flavonoid contents of different organs of eight accessions CG in the vegetative, flowering, and seed set stages.

Stage	Organ	TPC (mg GAE/g, DW)				TFC (mg RE/g, DW)			
		Min	Max	Range	Average	Min	Max	Range	Average
Vegetative	LS	24.56	37.58	13.02	29.56 ± 4.40 ^c^	251.43	325.89	74.46	284.72 ± 27.06 ^a^
Flowering	LS	18.23	34.37	16.14	26.23 ± 5.41 ^d^	191.29	282.93	91.64	229.95 ± 29.62 ^c^
	F	34.56	51.16	16.60	40.05 ± 5.28 ^b^	176.98	288.66	11.68	226.37 ± 36.12 ^c^
Seed set	LS	24.92	41.07	16.15	29.95 ± 5.73 ^c^	179.84	254.30	74.46	211.70 ± 30.77 ^d^
	S	34.83	108.86	74.03	60.27 ± 21.26 ^a^	191.29	417.53	226.24	272.19 ± 66.97 ^b^

The average is expressed as a mean value ± standard deviation equivalent; DW = extract dry weight; GAE = gallic acid equivalent, RE = rutin equivalent; LS = leaves and stem; F = flower; S = siliques. Values followed by the different letters in specific columns are significantly different (*p* < 0.05) according to Tukey’s multiple comparison test. Bolded values represent the highest content of the specific compound.

**Table 2:** Ability to scavenge free radicals expressed as RC_50_ values of CG accessions different organ extracts obtained at different growth stages.

	DPPH RC_50_ (µg/mL ± SD)					ABTS RC_50_ (µg/mL ± SD)				
Accession	LSV	LSF	LSS	FF	SS	LSV	LSF	LSS	FF	SS
TT-00	1090.87 ± 12.69 ^B^	1083.33 ± 9.4 ^A^	1405.96 ± 28.87 ^B^	716.54 ± 7.14 ^C^	565.93 ± 2.77 ^G^	226.39 ± 4.59 ^A^	301.29 ± 3.51 ^B^	337.51 ± 11.42 ^DE^	253.06 ± 3.37 ^BC^	136.02 ± 2.73 ^E^
UAG/1907C	925.95 ± 9.24 ^A^	1198.63 ± 11.58 ^B^	1598.41 ± 2.11 ^C^	753.83 ± 9.44 ^D^	721.45 ± 4.45 ^H^	283.23 ± 2.69 ^C^	295.19 ± 6.56 ^B^	399.47 ± 5.2 ^G^	283.94 ± 3.52 ^D^	224.98 ± 0.54 ^F^
ELG/1907C	994.04 ± 12.05 ^A^	1819.5 ± 8.57 ^F^	1382.04 ± 26.83 ^B^	674.86 ± 2.6 ^B^	345.30 ± 6.35 ^D^	366.81 ± 2.17 ^F^	472.95 ± 2.4 ^G^	357.41 ± 1.57 ^F^	243.95 ± 5.1 ^B^	96.17 ± 3.47 ^C^
ELG/1907B	1426.4 ± 17.29 ^E^	1297.93 ± 37.88 ^C^	1301.85 ± 17.14 ^B^	827.89 ± 3.02 ^E^	368.36 ± 0.08 ^E^	336.9 ± 1.4 ^E^	320.79 ± 0.88 ^C^	348.39 ± 3.19 ^EF^	289.1 ± 0.31 ^D^	91.24 ± 0.08 ^C^
WPK/2007	1247.02 ± 11.35 ^C^	1590.34 ± 12.37 ^E^	997.12 ± 30.59 ^A^	716.63 ± 2.76 ^C^	317 ± 2.49 ^B^	380.84 ± 3.23 ^G^	388.91 ± 10.6 ^E^	240.35 ± 2.07 ^B^	253.8 ± 1.75 ^BC^	92.52 ± 2.46 ^C^
KF-14	976.97 ± 3.88 ^A^	1877.51 ± 1.39 ^G^	1677.79 ± 125.27 ^C^	722.44 ± 4.72 ^C^	137.27 ± 7.1 ^A^	261.17 ± 4.45 ^B^	422.26 ± 9.36 ^F^	273.27 ± 8.21 ^C^	256.66 ± 6.75 ^C^	49.72 ± 0.41 ^A^
KF-05A	1466.595 ± 57.88 ^E^	1465.63 ± 23.2 ^D^	1312.18 ± 68.22 ^B^	822.11 ± 5.12 ^E^	388.21 ± 1.55 ^F^	359.1 ± 1.02 ^F^	337.64 ± 3.28 ^D^	330.01 ± 5.83 ^D^	295.26 ± 3.21 ^D^	107.43 ± 0.44 ^D^
KF-03	1333.84 ± 12.57 ^D^	1083.86 ± 0.94 ^A^	855.62 ± 7.59 ^A^	618.98 ± 6.58 ^A^	330.2 ± 3.98 ^C^	307.58 ± 1.27 ^D^	272.68 ± 0.96 ^A^	218.94 ± 0.49 ^A^	179.03 ± 4.63 ^A^	84.63 ± 2.25 ^B^
Average	1182.46 ± 17.12 ^c^	1427.09 ± 13.17 ^e^	1316.37 ± 38.33 ^d^	731.66 ± 5.17 ^b^	396.71 ± 3.60 ^a^	315.25 ± 2.60 ^c^	352.12 ± 4.31 ^d^	313.17 ± 4.75 ^c^	256.72 ± 3.58 ^b^	110.34 ± 1.55 ^a^

Data are presented as means ± standard deviation, *n* = 3, for each organ in each accession. The antioxidant activity was expressed as RC_50_ values, meaning that a higher value corresponds to lower antioxidant potential. RC_50_ = extracts concentration with 50 % antioxidant potential; LSV = leaves and stem in the vegetative stage; LSF = leaves and stem in the flowering stage; LSS = leaves and stem in the seed set stage; FF = flowers in the flowering stage; SS = siliques in the seed set stage. Mean values in the same column followed by the different capital letters indicate significant differences in the accessions RC_50_, while averages with different small letters indicate significant differences in the RC_50_ for the organs at *p* < 0.05.

**Table 3:** Content of rutin and astragalin (mg/g of the extract dry weight) in the extracts from the organs of CG accessions at vegetative, flowering, and at seed set stage.

	Rutin Content (mg/g, DW)					Astragalin Content (mg/g, DW)
Organ and Stage Accession	LSV	LSF	LSS	FF	SS	FF
TT-00	47.45 ± 1.98 a	33.57 ± 0.43 b	34.16 ± 1.41 c	75.50 ± 1.62 bc	32.78 ± 1.02 e	0.69 ± 0.04 b
UAG/1907C	33.10 ± 1.41 c	32.47 ± 1.10 b	26.78 ± 0.75 d	56.23 ± 0.63 e	30.61 ± 0.69 e	0.98 ± 0.18 b
ELG/1907C	37.59 ± 2.78 bc	33.40 ± 1.44 b	55.98 ± 0.98 a	74.17 ± 1.87 bc	49.84 ± 0.11 c	1.05 ± 0.34 b
ELG/1907B	41.49 ± 1.86 abc	34.67 ± 0.60 ab	45.78 ± 1.08 b	77.37 ± 0.46 bc	54.01 ± 0.27 b	2.02 ± 0.42 a
WPK/2007	36.22 ± 0.57 bc	25.81 ± 1.17 c	57.92 ± 0.79 a	71.24 ± 1.73 cd	45.66 ± 0.83 d	1.02 ± 0.27 b
KF-14	45.18 ± 1.86 ab	40.49 ± 0.80 a	59.13 ± 1.11 a	92.00 ± 1.21 a	79.72 ± 0.78 a	0.54 ± 0.26 b
KF-05A	40.74 ± 2.92 abc	35.51 ± 2.08 ab	53.55 ± 1.88 a	80.96 ± 2.15 b	42.02 ± 1.08 d	1.28 ± 0.41 ab
KF-03	37.49 ± 1.72 bc	31.89 ± 1.52 bc	43.34 ± 1.56 b	66.15 ± 0.27 d	54.03 ± 1.17 b	0.80 ± 0.13 b
Average	39.91 ± 5.40 C	33.48 ± 4.31 D	47.08 ± 11.44 B	74.20 ± 10.23 A	48.58 ± 14.74 B	1.05 ± 0.50 E

Data are presented as means ± standard deviation, *n* = 3, in each accession. LSV = leaves and stem in the vegetative stage; LSF = leaves and stem in the flowering stage; LSS = leaves and stem in the seed set stage; FF = flower in the flowering stage; SS = siliques in the seed set stage. Capital letters indicate significant variations in the average rutin content in organs across the different growth stages and small letters in specific columns indicate rutin variations among the accessions. Means in the same column followed by the different small letters indicate significant differences in the accessions rutin content, while averages with different capital letters indicate significant differences in the rutin content for the organs at *p* < 0.05.

## Data Availability

Data is contained within the article and supplementary material.

## Supplementary Materials

The following are available online at https://www.mdpi.com/article/10.3390/antiox10121952/s1, Figure S1: chromatograms of extracts obtained from leaves and stem organs of the eight CG accessions at the vegetative (A), flowering (B), and seed set stages (C). Figure S2: Chromatograms of extracts from flower organs of the eight CG accessions. Figure S3: Chromatograms of extracts from silique organs of the eight CG accessions. Figure S4: Correlation matrix plot showing Pearson’s correlation coefficient (*r*^2^) among the variables (phytochemicals (TPC, TFC, rutin), antioxidant activity, and extracts ability to scavenge free radicals).
