# Supplementary material for: Variation in Phenolic Compounds and Antioxidant Activity of Various Organs of African Cabbage (Cleome gynandra L.) Accessions at Different Growth Stages
Source: Antioxidants (Basel). 2021 Dec 6;10(12):1952. doi: 10.3390/antiox10121952 (PMC8750509; doi:10.3390/antiox10121952)
Supplement: Supplementary file 1 [file antioxidants-10-01952-s001.zip › antioxidants-1487053-supplementary.pdf]

A

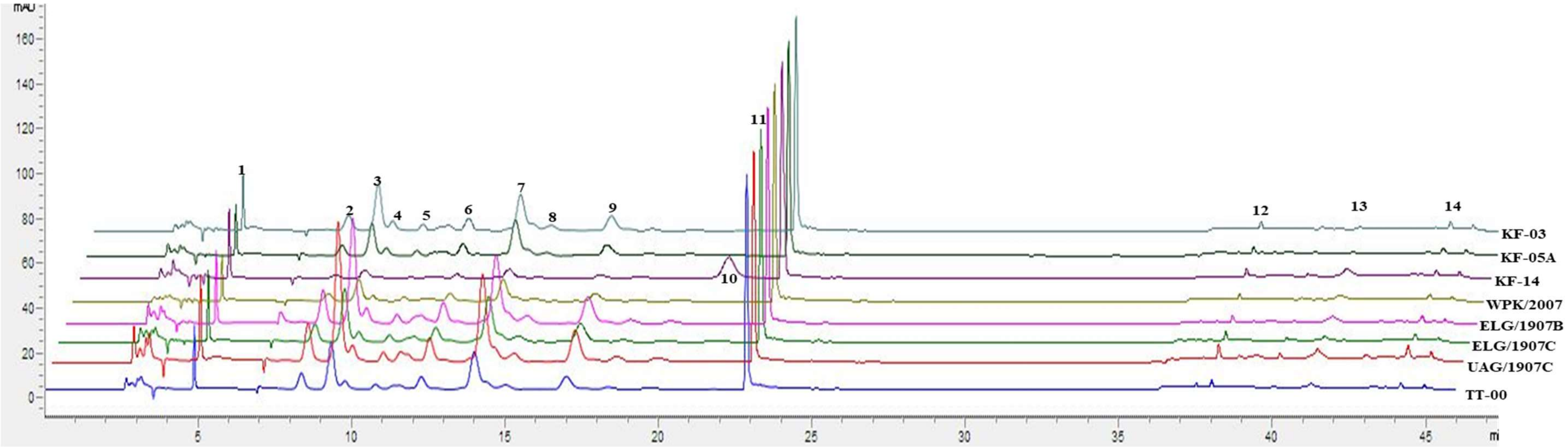
mAU
1
2
3
4
5
6
7
8
9
10
11
12
13
14
KF-03
KF-05A
KF-14
WPK/2007
ELG/1907B
ELG/1907C
UAG/1907C
TT-00

B

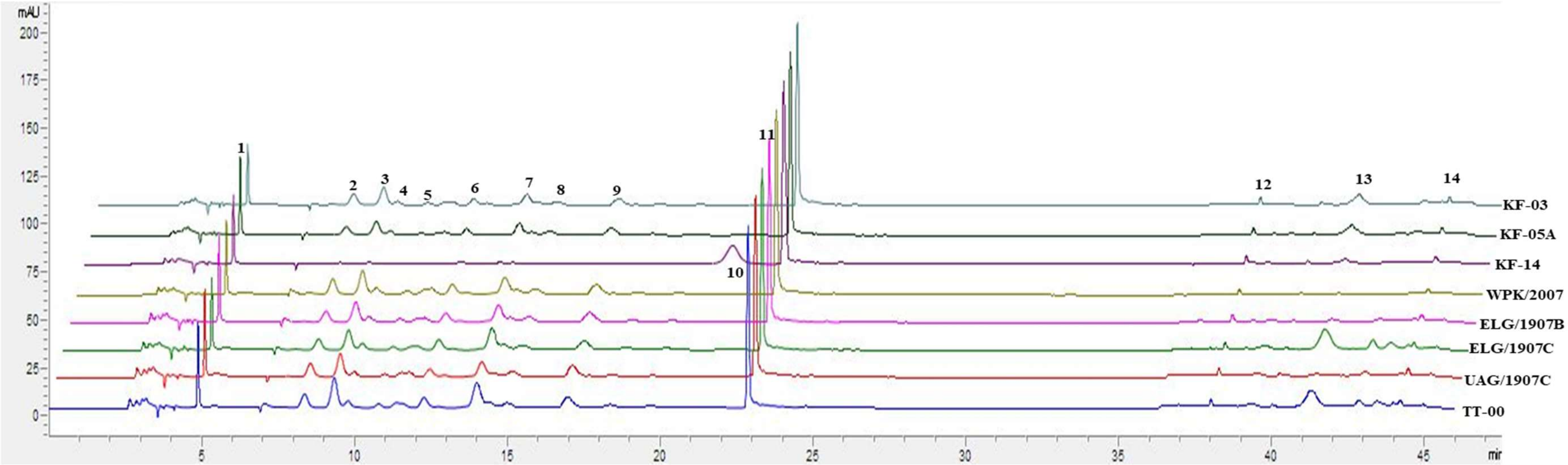
mAU
1
2
3
4
5
6
7
8
9
10
11
12
13
14
KF-03
KF-05A
KF-14
WPK/2007
ELG/1907B
ELG/1907C
UAG/1907C
TT-00

C

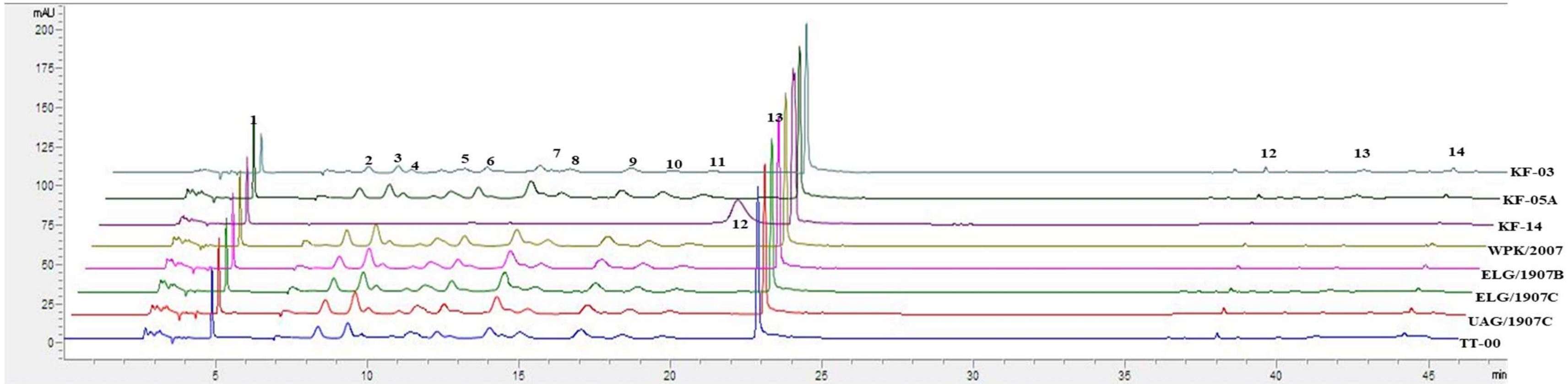

Supplementary Figure S1. LS (leaves and stem) organ extract chromatograms obtained at the vegetative (A), flowering (B), and seed set stages (C). Except for KF-14, which lacks some compounds (2-11) and has an extra compound (12) on its profile, the accessions show a similar pattern of phenolic compound profiles.

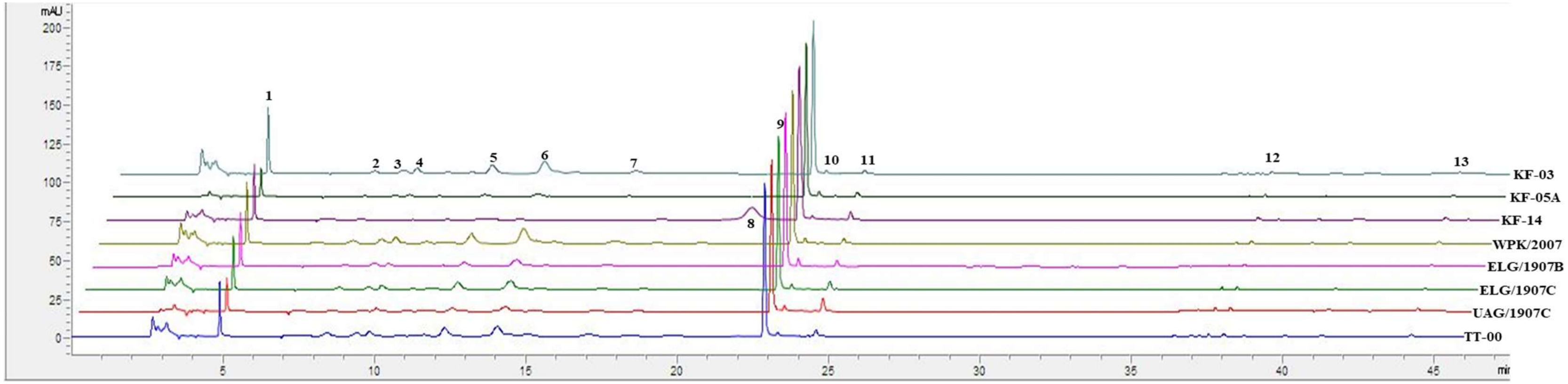

Supplementary Figure S2. The chromatograms of flower extracts revealed some differences in the compounds identified compared to the LS extracts. Except for KF-14, which has an extra compound 8 on its profile, the phenolic compound profiles of the accessions follow a similar pattern.

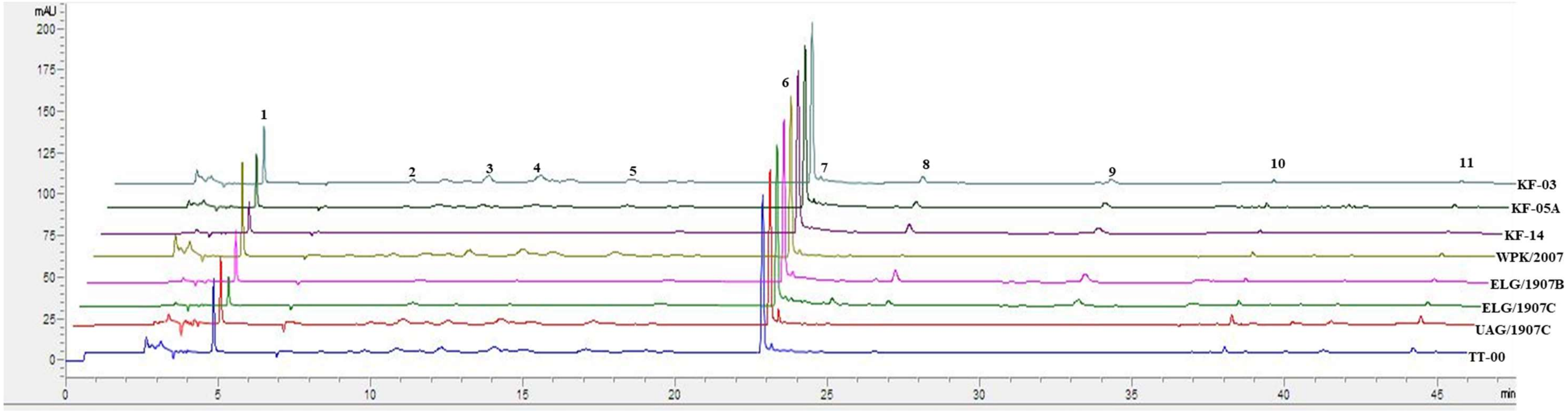

Supplementary Figure S3: The chromatogram of siliques extracts show different compound profiles compared to both LS and FF extracts. Siliques of accessions UAG/1907C lack compound 8 and 9 while those of TT-00 and WPK/2007 lack compound 9.

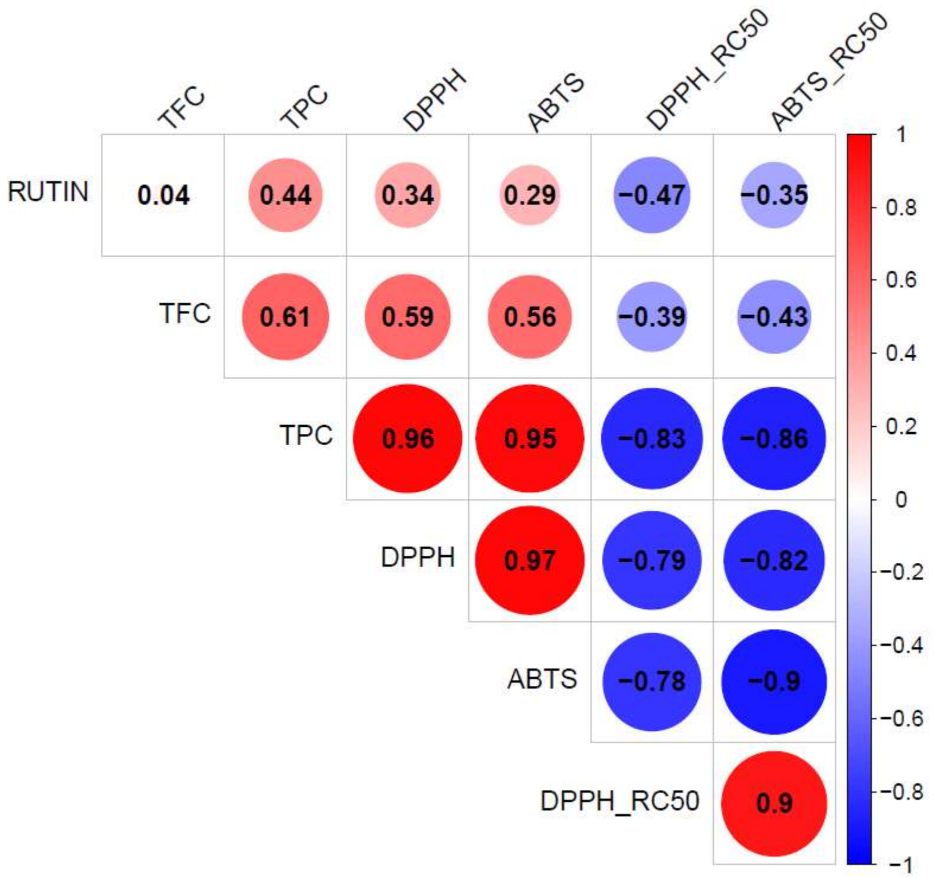

Supplementary Figure S4: Correlation matrix plot showing Pearson's correlation coefficient (r^2) among the variables (phytochemicals, antioxidant activity and extracts ability to scavenge free radicals). A strong positive correlation was found between the TPC, TFC and the DPPH and ABTS activities while rutin moderately contributed to the antioxidant activity.

ABTS = 2,2′-Azinobis-(3-Ethylbenzthiazolin-6-Sulfonic Acid), DPPH = 2,2-diphenyl-1-picrylhydrazyl, TPC = total phenolic contents, TFC = total flavonoid contents, RC_{50} = dose required to scavenge fifty percent of radicals.
